# Left Ventricle Segmental Longitudinal Strain and Regional Myocardial Work Index Could Help Determine Mitral Valve Prolapse Patients with Increased Risk of Ventricular Arrhythmias

**DOI:** 10.3390/jcdd10040181

**Published:** 2023-04-20

**Authors:** Ludmiła Daniłowicz-Szymanowicz, Agnieszka Zienciuk-Krajka, Elżbieta Wabich, Marcin Fijałkowski, Jadwiga Fijałkowska, Krzysztof Młodziński, Grzegorz Raczak

**Affiliations:** 1 Department of Cardiology and Electrotherapy, Faculty of Medicine, Medical University of Gdansk, 80-210 Gdansk, Poland; 2 I Department of Cardiology, Faculty of Medicine, Medical University of Gdansk, 80-210 Gdansk, Poland; 3 II Department of Radiology, Faculty of Health and Sciences, Medical University of Gdansk, 80-210 Gdansk, Poland

**Keywords:** mitral valve prolapse, nonsustained ventricular tachycardia, speckle tracking echocardiography, longitudinal strain, myocardial work

## Abstract

Mitral valve prolapse (MVP) could associate with malignant ventricular arrhythmias (VAs). Mitral annular disjunction, a putative mechanism for an arrhythmic substrate, leads to excessive mobility, stretch, and damage of some segments. Speckle tracking echocardiography (STE), with particular attention to the segmental longitudinal strain and myocardial work index (MWI), could be an indicator of the segments we aimed to check. Seventy-two MVP patients and twenty controls underwent echocardiography. Complex VAs documented prospectively after the enrollment was qualified as the primary endpoint, which was noticed in 29 (40%) patients. Pre-specified cut-off values for peak segmental longitudinal strain (PSS) and segmental MWI for basal lateral (−25%, 2200 mmHg%), mid-lateral (−25%, 2500 mmHg%), mid-posterior (−25%, 2400 mmHg%), and mid-inferior (−23%, 2400 mmHg%) segments were accurate predictors of complex VAs. A combination of PSS and MWI increased the probability of the endpoint, reaching the highest predictive value for the basal lateral segment: odds ratio 32.15 (3.78–273.8), *p* < 0.001 for PSS ≥ −25% and MWI ≥ 2200 mmHg%. STE may be a valuable tool for assessing the arrhythmic risk in MVP patients. Excessively increased segmental longitudinal strain with an augmented regional myocardial work index identifies patients with the highest risk of complex VAs.

## 1. Introduction

Mitral valve prolapse (MVP) is a common cardiac disease, well-characterized using echocardiography [1,2]. Most patients have a benign course; however, reports of sudden cardiac death (SCD) have been documented [3,4,5], raising a question about risk stratification in these patients. In the unselected MVP population, an annual SCD rate has been documented below 1% [6,7]. However, the prevalence of MVP at autopsy among young patients with SCD is up to 7% [8]. Although severe mitral regurgitation (MR), as well as its consequence on the left ventricle (LV) function and size, could predispose MVP patients to arrhythmic events [9,10], some individuals remain at a higher risk of SCD despite the absence of MR and LV dysfunction [9,10,11,12,13], which makes the group of patients interesting, but challenging due to the low event-rate. The underlying mechanisms of the arrhythmic MVP phenotype in patients without severe MR and LV dysfunction remain incompletely understood [6,14]. MVP patients are characterized by frequent ventricular arrhythmias (VAs), which are rarely severe [15]. However, fast nonsustained ventricular tachycardia (NSVT), sustained VT or ventricular fibrillation (VF) are known to be associated with excess mortality and should be taken into consideration in MVP patients [16]. Apart from the known demographic parameters or electrocardiographic changes (for instance, T-wave inversion or the presence of atrial fibrillation) [6,8], some morphological features seem to increase the risk of complex VAs in MVP patients [5,14]. Recently, the presence and severity of mitral annular disjunction (MAD), the potential mechanism responsible for VAs, have been studied intensively [17,18,19]. MAD is an abnormal systolic displacement of the hinge point of the mitral valve away from the LV myocardium wall [19], most frequently localized under the posterior mitral valve leaflet [16,20,21]. Despite that MAD could be present in healthy hearts [22,23,24,25], some authors revealed its connection with the arrhythmic the MVP phenotype [6,8,19,22,23,26,27]. MAD leads to the excessive mobility of the leaflets, accounting for a mechanical stretch of the inferobasal wall and papillary muscles, eventually leading to myocardial damage and fibrosis [16] as a possible substrate for VAs [28]. A robust technique that precisely assesses myocardium fibrosis is cardiac magnetic resonance (CMR) [3,29,30]. Since CMR is not a widespread diagnostic tool, finding noninvasive techniques easily performed in routine clinical practice, which could help identify MVP patients with a morphological substrate for VAs, is of great clinical importance [31]. Two–dimensional (2D) transthoracic echocardiography is unquestionably the first-step imaging modality for MVP diagnosis, and assessing its structural characteristics [16,23,32]. The length of the MAD, curling visualization, and the Pickelhaube sign are proposed to be the feature of the arrhythmic MVP phenotype in standard 2D echocardiography [33,34]. Two–dimensional speckle tracking echocardiography (STE) with segmental longitudinal strain may allow for precise evaluation of the myocardium regarding subtle changes [35]. Additionally, novel STE-derived myocardial work (MW) analysis helps estimate the regional left ventricle (LV) work by using strain values updated by overload conditions with formulating LV pressure-volume loops [35]. According to the data from the literature, longitudinal strain and MW parameters could be feasible indicators of future fibrosis appearance in LV segments with previously increased contractility [36]. Our study aimed to check whether 2D STE with segmental longitudinal strain and STE-derived MW analysis may allow for a more accurate assessment of the arrhythmic risk in MVP patients.

## 2. Materials and Methods

### 2.1. Study Design and Patient Population

From January 2017 to July 2021, consecutive patients treated in our University Arrhythmia Outpatient Clinic for different rhythm disturbances, in whom MVP was diagnosed using standard echocardiography [37], were enrolled in the study. The *exclusion criteria* were: patients with age < 18 years old, permanent or persistent atrial fibrillation/flutter, at least moderate valvular defects (including mitral regurgitation of moderate or more severe grades), congenital heart disease, previous valvular surgery, cardiomyopathy (hypertrophic, dilated or arrhythmogenic), or severe general conditions. Sex- and age-matched volunteers constituted the control group. Demographic and clinical characteristics were collected for every patient with a detailed past medical history. At the enrollment visit, echocardiography was performed, and a 24-h HOLTER-ECG was scheduled. Any complex VAs (nonsustained ventricular tachycardia (NSVT), sustained VT or VF) documented after the enrollment was qualified as the primary endpoint. The study protocol was approved by the local ethics committee of the Medical University of Gdansk (NKBBN/15/2021) and written informed consent was obtained from all participants.

### 2.2. HOLTER-ECG Monitoring

HOLTER-ECG monitoring, with detailed analysis of VAs, according to present recommendations [16] was performed in every patient after the enrollment visit. The number of premature ventricular contractions and their morphologies (monomorphic and polymorphic—three or more distinct morphologies), the presence of NSVT (defined according to EHRA recommendations [16] as three or more consecutive ventricular beats at a rate of >100 beats per minute lasting for 30s or less), or sustained VT or VF were noted.

### 2.3. Echocardiography

**Figure 1:**
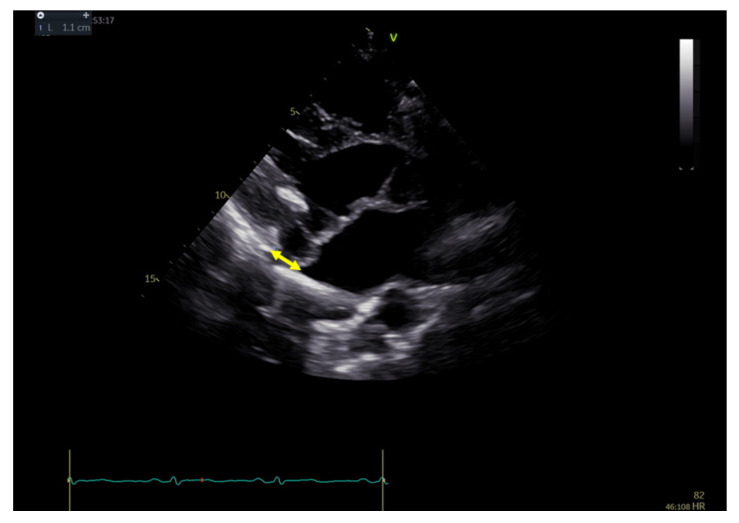
Transthoracic echocardiography (long-axis parasternal view) of MAD measurement in MAD+ patients. MAD distance is calculated as the length of systolic separation between the left atrial wall and mitral valve leaflet junction to the top of the left ventricle free wall.

**Figure 2:**
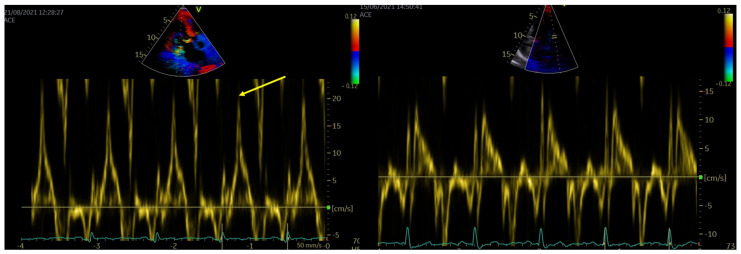
The presentation of the Pickelhaube sign (yellow arrow) as a spiked configuration of basolateral annular tissue Doppler imaging presentation (left panel). The presentation of S′ value of basolateral annular tissue Doppler imaging in patient without Pickelhaube sign (right panel).

**Figure 3:**
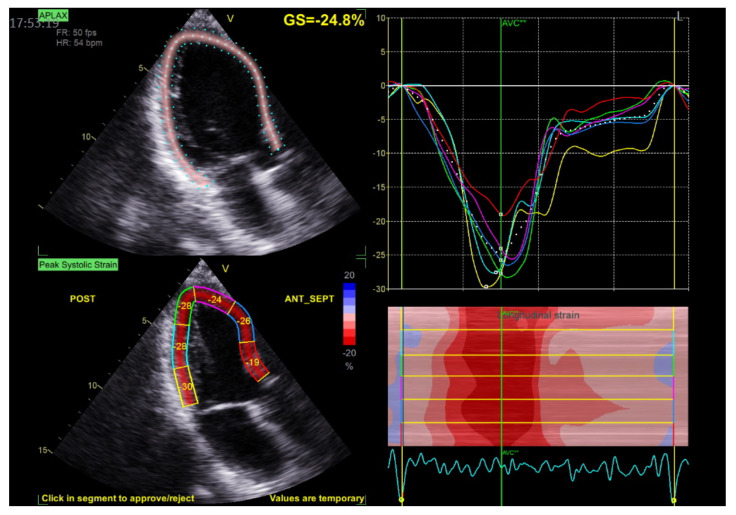
The example of representative case with speckle-tracking analysis including MAD area.

Each person underwent transthoracic echocardiography (GE VIVID E95, Horten, Norway). The blood pressure was measured in each patient just before echocardiographic examination. For each view, three consecutive heart cycles were recorded during quiet respiration; grayscale recordings were optimized at 50–80 frames/s, and only participants with these technical parameters were included for further analyses. All echocardiograms were stored digitally, and further offline analysis was performed using a commercial EchoPAC workstation (v204, GE Healthcare Horten, Norway). Standard echocardiographic parameters were obtained according to the principles described in the literature [38]. During end-systole, the MAD distance was measured from the left atrial wall and mitral valve leaflet junction to the top of the LV free wall in a parasternal long-axis view [33]. Figure 1 and the Supplementary Video demonstrate the example of the MAD presence and measurement. The Pickelhaube sign was noticed as a spiked configuration of basolateral annular TDI presentation (Figure 2). Patients were qualified for MAD+ or MAD− groups based on the presence of any-length MAD. Two-dimensional STE parameters were analyzed according to the appropriate recommendations [39]. For 2D longitudinal speckle tracking analysis, three endocardial markers were placed in an end-diastolic frame at apical four-, two-, and three-chamber views. The software automatically tracked the contour of the endocardium to cover the myocardial thickness of the entire LV wall. Adequate tracking was verified in real-time and corrected by adjusting the region of interest or manually correcting the contour to ensure optimal tracking. Due to the lack of particular recommendations regarding the MAD area examination, we included that in the STE analysis (Figure 3). Segments with low tracking quality were not considered for further STE analysis; if the patient had three or more segments with poor tracking quality, the software automatically did not allow further analysis, and those patients were excluded from further analysis. The 2D peak systolic longitudinal strain (global longitudinal strain—GLS) was analyzed from three apical views (4-, 2-, and 3-chamber views) and calculated for 16 from 17 segments (6 basal, 6 mid, and 4 apical). The longitudinal peak segmental strains were calculated for each segment [39,40,41]. Peak strain dispersion (PSD) was calculated automatically by the software as the standard deviation of each segment’s time to peak strain. MW parameters were quantified using the principles described in the literature [42,43,44]. The software calculates global constructive work (GCW), global wasted work (GWW), global work index (GWI), and global work efficiency (GWE) as the mean parameters from respective segmental values [45]. In our study, GLS, GCW, GWW, GWI, and GWE, as well as segmental calculations (for longitudinal Peak Segmental Strain and MW index (MWI)), were calculated and compared between the groups.

### 2.4. Statistics

Continuous data were presented as median (25th–75th percentile), whereas categorical data was presented as proportions. The Shapiro–Wilk test was performed to determine whether data were normally distributed. Most of the analyzed parameters did not have a normal data distribution, even after logarithmic data transformation. Therefore, we selected appropriate statistical analysis methods based on non-parametric tests. As appropriate, comparisons between groups were performed with the Mann–Whitney U-test for continuous variables and Pearson’s chi-square test for categorical variables. The accuracy of pre-specified echocardiographic cut-off values as potential predictors of the documented NSVT was determined based on the area (AUC) under the receiver-operating characteristic (ROC) curve: the Youden index was calculated as sensitivity + specificity −1, and the cut-off values were derived by maximalization of the sum of sensitivity and specificity; the parameters with AUC values higher than 70% were taken into consideration for further analyses. The logistic regression analysis assessed an association between the analyzed parameters (with pre-specified in ROC analysis cut-off values) and the endpoint. Intra- and inter-observer reproducibility of all segmental strain parameters and segmental MWI values was assessed on 20 randomly selected patients: the intra-class correlation coefficient (ICC), coefficient of variation (CV), lower and upper limits of agreement, and mean bias (Bland-Altman test) were calculated. The clinical significance of the ICC was interpreted as follows: excellent, ICC ≥ 0.80; good, 0.60 ≤ ICC < 0.80; moderate, 0.40 ≤ ICC < 0.60; and poor, ICC < 0.40 [45]. Values of *p* < 0.05 were considered significant. The statistical analysis was conducted with the R 3.1.2. environment (R Core Team, Vienna, Austria).

## 3. Results

### 3.1. Baseline Characteristics

**Figure 4:**
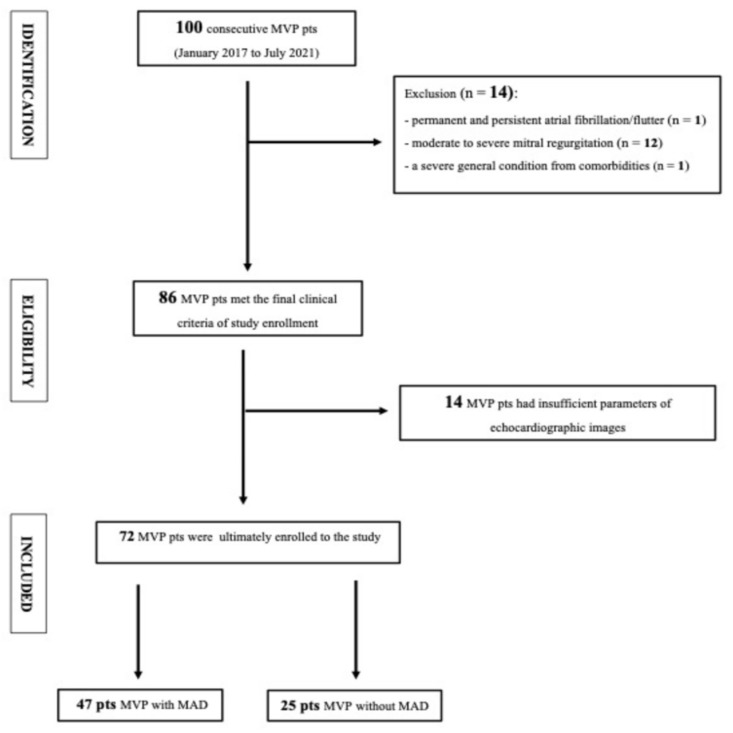
Flow chart of screened, included and excluded MVP patients.

From 100 screened patients with MVP, 86 were initially enrolled in the study based on inclusion and exclusion criteria. Fourteen patients were further excluded due to inappropriate echocardiographic quality and thus we were unable to perform precise 2D STE analysis. Finally, 72 patients were considered for further calculations (Figure 4).

The mean age of the MVP patients was 40 (33–49 years old), and 51 (71%) patients were female. Most of them (67%) complained about palpitations and were treated with beta-blockers (72%). In the previous history of the enrolled patients, seventeen (24%) had documented (at the span of 23 years before the enrollment) sudden cardiac arrest due to idiopathic VF, from which 15 had implanted ICDs in the secondary SCD prevention (2 patients after sudden cardiac arrest refused ICD implantation). Two patients without sudden cardiac arrest had ICDs implanted in primary prevention: one patient with LQTS type 2 after syncope while on adequate beta-blocker therapy and the second with LV hypertrabeculation and frequent sudden cardiac arrest cases in the family history. Table 1 presents the clinical characteristics of the MVP patients.

Based on the presence of any-length MAD, 47 patients were qualified as MAD+ and 25 as MAD− patients. The MAD+ group was younger (with borderline statistical significance) and characterized by significantly more frequent palpitations than MAD− patients (Table 2).

Within one month after the enrollment, all patients had 24-h HOLTER-ECG monitoring. The primary endpoint was noticed in 29 (40%) patients, which in all cases the NSVTs were without other complex ventricular arrhythmias. From all patients with implanted ICDs, 14 (82%) of them had PVCs in the 24-h HOLTER monitoring, and in 12 (71%) of them NSVT was observed (from which 10 patients had polymorphic pattern of NSVT).

NSVTs were observed significantly more often in MAD+ patients, contrary to MAD−. Additionally, MAD+ patients were characterized by significantly faster NSVTs (Table 3).

### 3.2. Echocardiography Parameters

Table 4 presents precise echocardiographic characteristics of the studied MVP patients compared to the healthy volunteers, and between MAD+ and MAD− patients. Median values of systolic blood pressure were 120 (120–123) mmHg and for diastolic was 70 (70–76) mmHg. Although the average values for all standard echocardiographic parameters were within the normal range, the whole group of MVP patients differed significantly from the controls regarding LA, LV, and RV size, as well as LVEF. MAD+ patients were characterized by similar changes contrary to healthy, while the differences between MAD+ and MAD− groups were not so prominent. Every MAD+ patient had a systolic curling phenomenon without its presence in the MAD− group. TDI parameters (S’ lat and S’ sept) were significantly higher in MAD+ patients compared to MAD− and healthy groups, with particularly prominent S’ lat with the Pickelhaube sign (Figure 3) found in 30 MAD+ patients and none in the MAD− group. The prevalence of the bileaflet variant of prolapse and mild mitral regurgitation were significantly higher in the MAD+ group vs. MAD−, while there were no statistical differences in the Barlow disease occurrence between the two groups. Within 2D STE calculations, MVP patients had similar GLS values as healthy persons (−22 (−23–−10)% vs. −22 (−22–−20)% respectively, *p* = 0.455), while LVEF was significantly lower (57 (54–61)% vs. 62 (60–65)% respectively, *p* < 0.000). At the same time, GLS was significantly augmented (more negative) in MAD+ (−22 (−23–−21)%) contrary to the MAD– group (−20 (−22–19)%, *p* = 0.027) with no differences regarding LVEF (56 (54–61)% vs. 58 (54–62)% respectively, *p* = 0.275). However, the GLS absolute values were similar between MVP and healthy persons; PSD values were significantly higher in MVP patients (44 (34–54) ms vs. 34 (27–38) ms respectively, *p* = 0.005), and in comparisons between each MVP group and control (Table 4).

**Figure 5:**
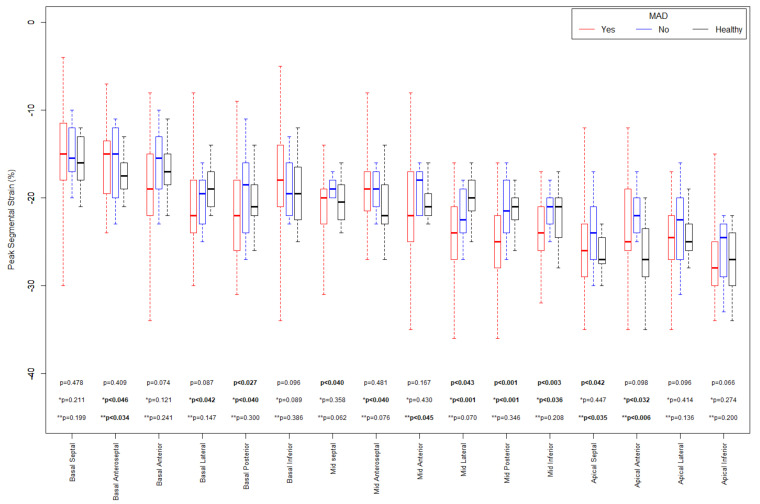
Peak Segmental Strain comparisons between MAD+, MAD− and Healthy groups in sixteen segments.

**Figure 6:**
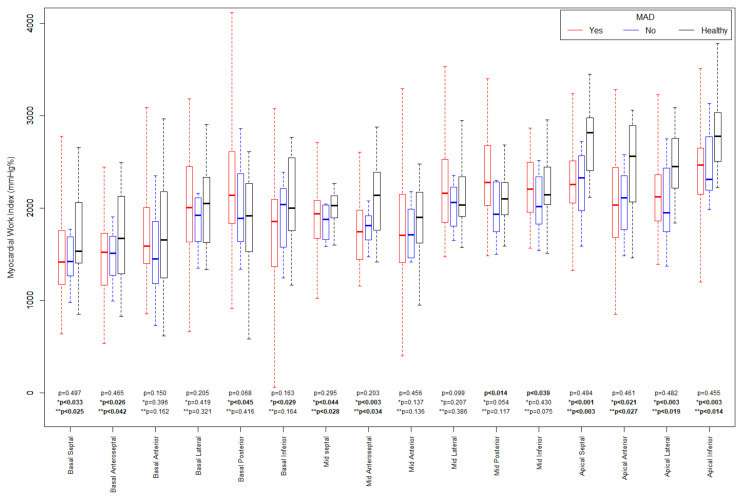
Myocardial Work Index comparisons between MAD+, MAD− and Healthy groups in sixteen segments.

**Figure 7:**
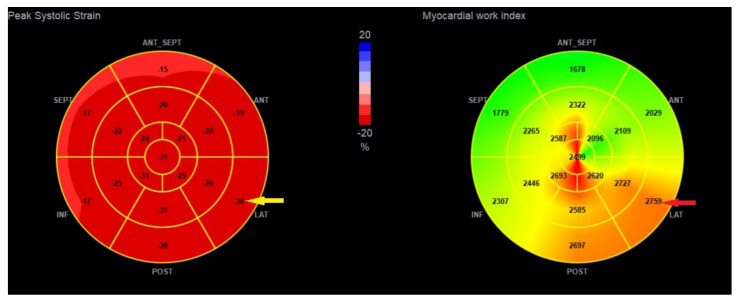
The example of the “Bull’s-eye” representation (obtained from long-axis, 2- and 4-chamber apical views) of regional strains in the MAD+ patient. Arrows present basal lateral segment with augmented (more negative) values of longitudinal strain (yellow arrow) and myocardial work index (red arrow).

**Figure 8:**
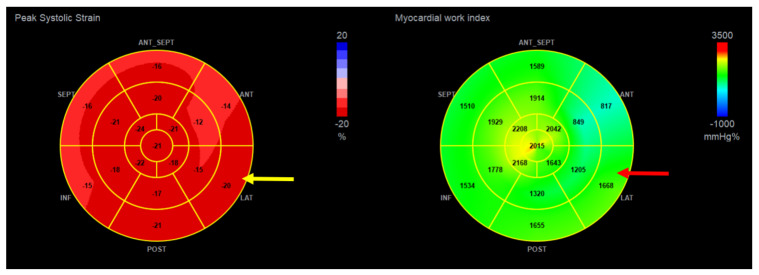
The example of the “Bull’s-eye” representation (obtained from long-axis, 2- and 4-chamber apical views) of regional strains in the MAD− patient. Arrows present basal lateral segment with normal (not augmented) values of longitudinal strain (yellow arrow) and myocardial work index (red arrow).

**Figure 9:**
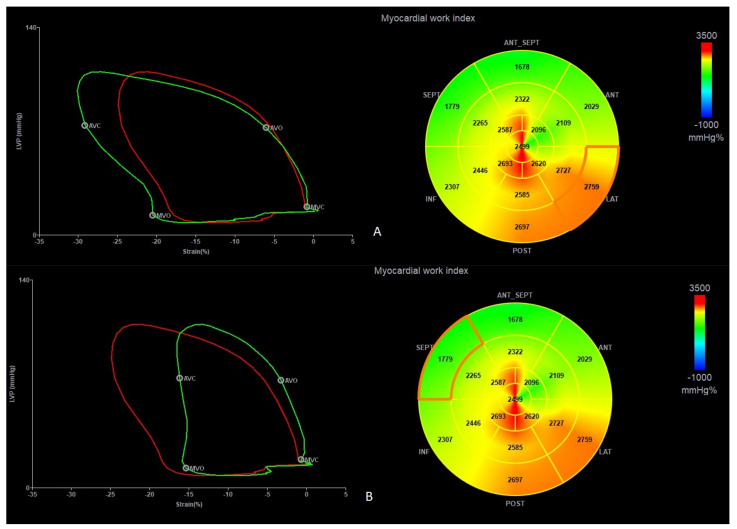
The example of the “Bull’s-eye” representation (obtained from long-axis, 2- and 4-chamber apical views) of regional strains in the MAD+ patient. Panel (**A**) presents the basal lateral segment with augmented values of longitudinal strain and MWI (marked segment). Panel (**B**) present basal septal segment with lower values of longitudinal strain and myocardial work index (marked segment).

A more detailed analysis regarding the segmental parameters between MAD+, MAD−, and healthy groups revealed many differences were observed regarding segmental 2D STE parameters, particularly with increased (more negative) peak segmental strain values in the MAD+ group in basal posterior and mid (posterior, lateral and inferior) segments and increased MWI values in MAD+ group in mid posterior and inferior segments (Figure 5 and Figure 6). Figure 7, Figure 8 and Figure 9 present the example of a “Bull’s-eye” representation (obtained from long-axis, 2- and 4-chamber apical views) of regional strains in the MAD+ and MAD− patients with particular attention to segments with augmented values of the MWI. Moreover, MAD+ patients had more segments with augmented (more negative than −25%) peak segmental strains than MAD− or healthy persons (Supplementary Table S1). The results of inter- and intra-observer variability show excellent results for peak segmental strains and the MWI with a slightly lower CV for the MWI (Supplementary Table S2).

### 3.3. Predictors of Arrhythmic Episodes

**Figure 10:**
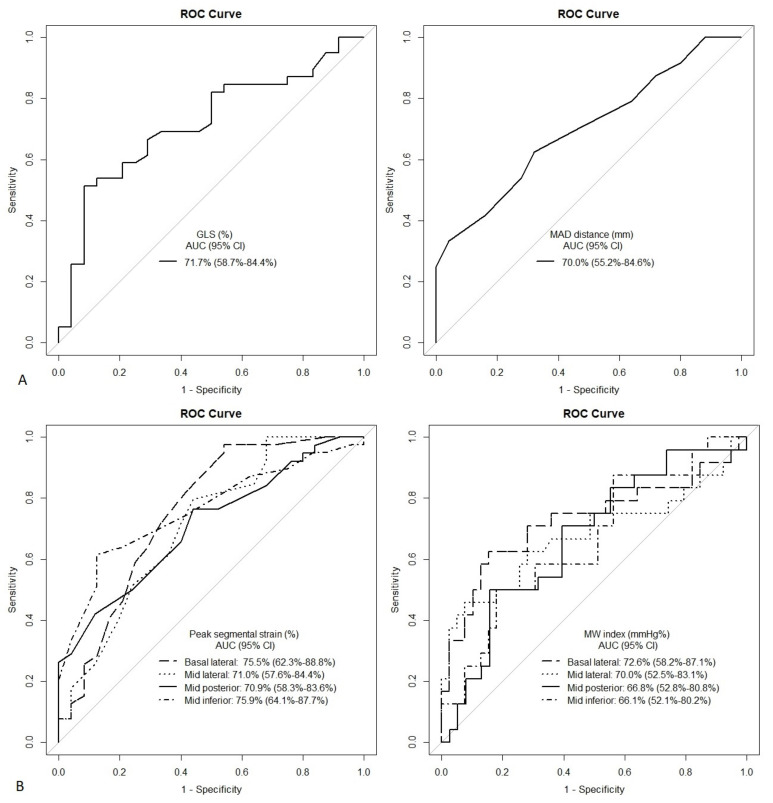
ROC curves of 2D GLS and MAD distance (panel (**A**)) and 2D peak segmental strain and segmental MWI parameters (panel (**B**)).

As we noted, the primary endpoint was noticed in 29 (40%) patients, in which all cases NSVTs were without other complex ventricular arrhythmias. Analysis of the ROC curve for the 2D STE parameters identified GLS, MAD distance, peak segmental strain for basal (lateral) and mid (lateral, posterior, and inferior) segments, and the MW index for basal lateral as the accurate predictors of the primary endpoint in the studied group of patients with AUC value higher than 70% (Table 5). The MW indexes for mid-lateral, mid-posterior, and mid-inferior segments were the accurate predictors of the complex VAs presence, with AUC values from 65 to 70%. Figure 10 presents ROC curves for MAD distance, GLS, peak segmental strains, and segmental MWI parameters. Other echocardiographic parameters were characterized by the lower discriminatory power and were not considered for further calculations. All mentioned parameters with adequate AUCs, calculated with pre-specified cut-off values, were significant predictors of NSVT in the univariate logistic regression analysis with higher OR for 2D STE parameters than for MAD (Figure 10); moreover, sensitivity was better for the segmental STE parameters in comparison to the GLS and MAD distances (Table 5).

A combination of peak segmental strain and MW index for the abovementioned segments increased the probability of complex VAs, revealing the highest value for the basal lateral segment (Table 6).

## 4. Discussion

The main finding of our study is that excessively increased (more negative) segmental longitudinal strain and a high MWI in basal and mid segments could help identify patients with an increased risk of complex VAs. To the best of our knowledge, this is the first study on the relationship between 2D STE with additional STE-derived MW analysis and arrhythmic risk in this group.

Several studies were dedicated to revealing potential predictors of life-threatening arrhythmias in MVP patients [8,14,16,17,22,26,27,33,34]. MAD, as an accessible echocardiographic parameter, has been considered one of the central morphologies as it correlates with VAs [17,33,46]. Therefore, in the first step of our study, we analyzed our patients according to the presence of this morphological feature, showing that MAD+ patients were characterized by the more frequent occurrence of different VAs (Table 2). Due to the low specificity and possibly benign character [15], we did not qualify premature ventricular contractions but complex VAs as the possible endpoint. Our data regarding the prognostic role of MAD distances in prediction complex VAs confirmed the previously published data with similar cut-off values for this parameter [17,18,19,20]; however, its sensitivity and specificity in our population were too low (Table 5). It is of note that MAD length measured by different methods and techniques is not interchangeable and should be interpreted with caution [17,47]. That can be explained by the morphological complexity and variability of the MAD area and localization, and the inferiority of standard echocardiography when diagnosing MAD compared to CMR [31]. On the other hand, it is unclear whether MAD presence is the only sufficient arrhythmic risk factor in MVP patients. Some authors emphasize that not only the MAD presence but significant hypercontractility of segments adjacent to MAD could be primary triggers for fibrotic foci formation, creating a substrate for VAs [6,34,48]. Moreover, our results suggest that MAD as a single factor could not be relevant to the genesis of augmented peak segmental strains, and probably other parameters, such as the size and area of MVP, may play a significant role; however, it is difficult to measure precisely by echocardiography.

Since the MAD area provokes excessive mobility, mechanical stretch, and possibly myocardial damage, this could be determined by STE. Therefore, our efforts were finally devoted to a more accurate assessment of STE (including segmental strains and MWI) as a possible correlation to complex VAs. The first observation of such myocardial hypermobility was described by Nutter et al. in an angiography examination [49]. Based on this hypothesis, the STE technique could play an essential role in the regional assessment of myocardial hypercontractility. For instance, Ermakov et al. showed an increase in the PSD value, due to local myocardium hypermobility, in MVP patients with VAs and indicated that the parameter was the only significant predictor of arrhythmic risk on multivariate analysis (OR 1.1, 95% CI 1.02 to 1.11, *p* = 0.006) [50]. In our study, PSD was significantly higher in MVP patients compared to healthy controls (44 (34 54) and 34 (27–38) ms, respectively, *p* = 0.005), and a little higher in MAD+ compared to MAD− persons (46 (36–56) and 38 (31–46) ms respectively, *p* = 0.051). However, that parameter did not have adequate discrimination power in the ROC analysis. Different exclusion criteria could explain the discrepancies with the results of Ermakov et al. [50]. Unlike in the cited study, we excluded patients with higher than mild mitral regurgitation due to its possible impact on the strain and MW values. Our decision to exclude patients with moderate and severe mitral regurgitation could omit some MVP patients with high arrhythmic risk due to possible hemodynamic consequences of regurgitation [50]; however, in the present study, we aimed to check the direct influence of abnormal, non-homogenous contractility of the myocardium, measured by 2D STE parameters, on arrhythmic risk, trying to exclude the possible effect of other factors. Based on Ermakov’s [50] and our results regarding the comparison of GLS between the MVP and healthy controls, and between MAD+ and MAD− patients, we hypothesized that the parameters of the regional longitudinal strain and their derivatives (i.e., MW parameters) could be of use in determining areas of increased contractility as opposed to GLS values. While GLS can be preserved or slightly altered in patients with MVP, some differences in segmental longitudinal strain peaks may be observed [51]. In our study, the GLSs were within the normal range in the MVP group and similar to healthy persons (−22 (−23–−10) and −22 (−22–−20)%, respectively, *p* = 0.445). At the same time, the longitudinal strains for particular segments were significantly different (Figure 5). That aspect was initially studied by van Wijngaarden et al., where it was demonstrated that some basal segments of the LV were characterized by regional hypermobility with higher (more negative) regional longitudinal strain; however, in that study, the relationship between increased strain values and the risk of VAs was not addressed [52], which is what we additionally checked in our study.

The connections between data presented in our study showed an increased shortening at segments correlating with papillary muscle insertion areas in MVP patients and VAs risk could be unique and opposed to other pathologies, such as HCM, Fabry disease, or cardiac amyloidosis. In these diseases, cardiac fibrosis, known as the main substrate of VAs, results from the specificity of these diseases and is not preceded by an increase in segmental contractility. In MVP patients, MAD causes excessive leaflet mobility, leading to hypercontractility and mechanical stretch in neighboring areas as a possible trigger of fibrotic foci formation. As a great exec, Perazzolo Mara M. et al. proves the connection between abnormal contractility produced by MAD with the mechanical stretch transmitted to papillary muscles leading to further possible occurrence of fibrosis in stretched areas (which was quantified by LGE areas in CMR) [17]. Vaidya et al. showed that surgical correction of MVP excluded the mechanical stretch and led to the significant reduction of VAs, what seems to support the theory of myocardial tissue damage due to hypercontractility and stretching [53]. Additionally, the historical study conducted by Franz et al. in 1992, using rabbit hearts, demonstrated that VAs were more likely to occur when the heart experiences a rapid myocardial stretch [54]. Researchers also concluded that in areas of the heart that experience greater stretch may serve as “foci” for stretch-activated arrhythmias during dynamic ventricular loading. The results from that study provide evidence for the existence of stretch-activated membrane channels in the ventricular myocardium, and may help to explain instances of ventricular ectopy in conditions, such as MVP syndrome, where there is a differential ventricular loading or regional muscle traction. From the molecular point of view, Morningstar et al. in their study conducted a histopathologic examination of biopsies taken from peripapillary muscles in patients who had undergone surgical mitral valve repairs [55]. Their findings suggest that the mechanical stress induced by prolapsing valves affects various types of cells, leading to the release of substances that promote fibrosis. Immunohistochemistry confirmed a significant increase of the collagen type I protein in these regions, and ultrastructural studies revealed a deficiency of primary cilia in areas with regional fibrosis, supporting the theory of mechanical stress impacting fibrosis pathways.

MW analysis used in the present study is a promising novel technique that helps to estimate the regional LV work using strain values updated by overload conditions with formulating LV pressure–volume loops [56]. It is possible to adulterate the accurate LV contractility using only GLS values, irrespective of the LV afterload conditions. Therefore, the combination of longitudinal strains with afterload conditions, as is estimated in MW analysis, seems to be more accurate in evaluating the work of the separate segments [16,57]. The physiological remodeling of a healthy heart due to increased afterload during physical activity could be accompanied by increased absolute values of longitudinal strain and MW, which is, however, more homogenous than in MVP patients, and presents only during physical exercise, and does not lead to abnormal stretching [58]. In MVP patients, significantly increased regional longitudinal shortening with increased MW values present permanently, i.e., also at resting conditions, and may result in oxidative stress and possible fibrotic response [57]—a potential substrate for VA.

Our study linked a combination of peak segmental strain and MWI for basal lateral and three mid (lateral, posterior, inferior) LV segments to the increased probability of complex VAs occurrence (Table 6). However, considering the current scarcity of data, further research is needed to verify this data’s clinical utility and predictive value.

## 5. Study Limitations

Our study has several limitations. First, the patients were recruited from our tertiary arrhythmia center. Thus, the incidence of different arrhythmias in those patients could be higher than expected from the general MVP population (what is confirmed in the number of retrospective SCA events); therefore, our findings reflect abnormalities found in arrhythmic MVP patients rather than being representative of typical MVP patients. Due to the necessity of selecting a clinically homogeneous group of patients, a single-center analysis with a relatively small group of patients is also limited by the requirement of adequate echocardiographic image quality. The small number of patients translated to the low number of complex VAs that were unable to perform adequate multivariate statistics, which would undoubtedly add to the value of our analysis. Next, omitting MVP patients with more than a mild mitral regurgitation could exclude some essential groups with high arrhythmic risk; however, that allowed us to avoid the possible influence of mitral regurgitation on the arrhythmic events and GLS and MWI parameters. Next limitation was due to the LVEF calculation by a biplane method, which, due to the systolic deflection of the MAD area, may reduce the end-systolic volume, increasing the real LVEF in MVP patients. Next, we did not analyze circumferential and radial strains, and the STE analysis was limited to the longitudinal strain as the most reproducible and quick-to-analyzer parameter.

## 6. Conclusions

Excessively increased segmental longitudinal strain with augmented regional MWI could discriminate the MVP patients with the highest risk of complex ventricular arrhythmias. Two-dimensional STE could be a valuable tool for characterizing MVP patients’ arrhythmic phenotype, encouraging further studies of this issue.

## Figures and Tables

**Table 1:** Clinical characteristics of MVP patients.

	MVP All n = 72
Age (years old)	40 (33–49)
Female sex, n (%)	51 (71)
**Previous medical history and clinical data**	
Palpitations, n (%)	48 (67)
Presyncope, n (%)	13 (18)
Syncope n (%)	10 (14)
History of sudden cardiac arrest, n (%)	17 (24)
Sudden cardiac death in a family history, n (%)	4 (6)
Implantable cardioverter-defibrillator, n (%)	17 (24)
Hypertension, n (%)	6 (8)
Coronary artery disease, n (%)	1 (1)
Hiperlipidemia, n (%)	4 (6)
Atrial fibrillation/flutter, n (%)	3 (4)
**Medications**	
Beta-blockers, n (%)	52 (72)
Angiotensin converting enzyme inhibitors/sartans, n (%)	1 (1)
Spironolactone, n (%)	6 (8)
Cordarone/Sotalol/Propafenon, n (%)	6 (8)
Diuretics, n (%)	1 (1)
Statins, n (%)	5 (7)

Continuous data are presented as median (25th–75th percentile), categorical as proportions. MVP: mitral valve prolapse.

**Table 2:** Clinical characteristics of MVP patients according to MAD presence.

	MAD (+) n = 47	MAD (−) n = 25	*p*
Age (years old)	39 (32–46)	41 (37–52)	0.074
Female sex, n (%)	34 (72)	17 (68)	0.787
**Previous medical history and clinical data**			
Palpitations, n (%)	37 (79)	11 (44)	0.004
Presyncope, n (%)	11 (23)	2 (8)	0.196
Syncope n (%)	9 (19)	1 (4)	0.149
History of sudden cardiac arrest, n (%)	12 (26)	5 (20)	0.773
Sudden cardiac death in a family history, n (%)	3 (6)	1 (4)	1.000
Implantable cardioverter-defibrillator, n (%)	13 (28)	4 (16)	0.384
Hypertension, n (%)	5 (11)	1 (4)	0.658
Coronary artery disease, n (%)	1 (2)	0 (0)	1.000
Hiperlipidemia, n (%)	1 (2)	3 (12)	0.117
Atrial fibrillation/flutter, n (%)	2 (4)	1 (4)	1.000
Beta-blockers, n (%)	35 (75)	17 (68)	0.589
Angiotensin converting enzyme inhibitors/sartans, n (%)	1 (2)	0 (0)	1.000
Spironolactone, n (%)	5 (11)	1 (4)	0.658
Cordarone/Sotalol/Propafenon, n (%)	5 (11)	1 (4)	0.658
Diuretics, n (%)	0 (0)	1 (4)	0.347
Statins, n (%)	2 (4)	3 (12)	0.334

Continuous data are presented as median (25th–75th percentile), categorical as proportions. MAD: mitral annular disjunction; MVP: mitral valve prolapse.

**Table 3:** The results of HOLTER-ECG monitoring performed within one month after the enrollment.

	MAD+ n = 47	MAD− n = 25	*p*
Heart rate mean (minimal–maximal) (bpm)	74 (63–83)	70 (67–76)	0.4904
Presence of any ventricular arrhythmias, n (%)	43 (91)	13 (52)	<0.001
Premature ventricular contractions (average number)	2600 (937–5250)	48 (7–752)	0.002
NSVT presence, n (%)	25 (53)	4 (16)	0.002
NSVT—cycle length (ms)	200 (177–254)	371 (297–411)	0.035
Polymorphic ventricular arrhythmia	22 (47)	0 (0)	<0.001

Continuous data are presented as median (25th–75th percentile), categorical as proportions. MAD: mitral annular disjunction; MVP: mitral valve prolapse, NSVT: non-sustained ventricular tachycardia.

**Table 4:** Echocardiographic characteristics of MVP patients.

	MVP n = 72	Healthy n = 20	*p*	MAD+ n = 47	MAD− n = 25	*p* *	*p* **	*p* ***
Bileaflet	48 (67%)	−	−	37 (79%)	11 (44%)	0.026	−	−
Barlow disease	11 (26%)	−	−	10 (21%)	1 (4%)	0.068	−	−
Trivial regurgitation	36 (50%)	−	−	19 (40%)	17 (68%)	0.043	−	−
Mild regurgitation	33 (46%)	−	−	24 (51%)	9 (36%)	0.232	−	−
LAV index (ml/m^2^)	29 (23–40)	20 (18–23)	<0.000	34 (23–40)	25 (22–30)	0.051	0.001	0.022
LVEDd (mm)	49 (46–54)	46 (41–46)	<0.000	50 (47–54)	49 (46–51)	0.074	<0.000	0.006
LVESd (mm)	34 (29–38)	28 (26–30)	<0.000	35 (29–39)	34 (29–37)	0.363	0.001	0.001
e′ (cm/s)	12 (10–14)	14 (10–16)	0.084	12 (10–14)	13 (10–14)	0.482	0.065	0.080
E/e′	5.9 (4.9–6.9)	6.3 (5–7.6)	0.233	5.9 (5.0–6.8)	5.9 (4.9–8.0)	0.418	0.267	0.409
S′ lat (cm/s)	12 (8–18)	8 (7–9)	<0.000	16 (12–22)	8 (7–10)	<0.000	<0.000	0.064
S′ sept (cm/s)	9 (8–10)	8 (8–9)	0.088	9 (8–11)	8 (7–8)	<0.002	<0.018	0.202
Pickelhaube sign, n (%)	30 (42%)	-	-	30 (64%)	-	-	-	-
RVID (mm)	36 (33–40)	26 (23–29)	<0.000	36 (33–39)	35 (33–41)	0.492	<0.000	<0.000
LVEF (%)	57 (54–61)	62 (60–65)	<0.000	56 (54–61)	58 (54–62)	0.275	<0.001	<0.004
GLS (%)	−22 (−23–−10)	−22 (−22–−20)	0.445	−22 (−23–−21)	−20 (−22–−19)	<0.027	0.128	<0.036
PSD (ms)	44 (34–54)	34 (27–38)	0.005	46 (36–56)	38 (31–46)	0.051	<0.002	0.064
GWI (mmHg%)	2001 (1775–2206)	2127 (2073–2354)	0.018	2052 (1811–2202)	1910 (1754–2200)	0.214	<0.028	<0.009
GCW (mmHg%)	2112 (1823–2324)	2145 (2006–2392)	0.099	2241 (1874−2363)	1874 (1797–2204)	0.064	0.235	<0.037
GWW (mmHg%)	124 (83–192)	143 (95 −188)	0.268	129 (87–197)	114 (79–163)	0.199	0.349	0.179
GWE (%)	94 (91–96)	94 (90–95)	0.403	93 (90–96)	94 (92–96)	0.278	0.482	0.278

Continuous data are presented as median (25th–75th percentile), categorical as proportions. *p*: *p* between MVP and Healthy; *p* *: *p* between MAD+ and MAD-; *p* **: *p* between MAD+ and Healthy; *p* ***: *p* between MAD- and Healthy. e′: early diastolic mitral myocardial peak velocity averaged from the lateral and septal positions, E/e′: the ratio between E and e′, GCW: global constructive work, GLS: global longitudinal strain of the left ventricle, GWE: global work efficiency, GWI: global work index, GWW: global wasted work, LAV index: indexed left atrial volume, LVEDd: left ventricular diastolic diameter, LVESd: left ventricular systolic diameter, LVEF: left ventricle ejection fraction, PSD: peak strain dispersion, RVID: Right ventricle internal diameter, S′ lat: peak lateral systolic velocities of mitral annulus, S′ sept: peak septal systolic velocities of mitral annulus.

**Table 5:** Cut-off values and prognostic accuracy of the analyzed parameters as predictors of the primary end-point.

	Cut-off	AUC%	Sensitivity	Specificity	PPV	NPV	OR	*p*
GLS (%)	−20	71.7 (58.7–84.40)	0.51	0.92	0.91	0.54	11.6 (2.39–56.1)	<0.001
MAD distance (mm)	10	70.0 (55.2–84.6)	0.62	0.68	0.65	0.65	3.54 (1.09–11.51)	0.042
Peak segmental strain (%)								
Basal lateral	−25	75.5 (62.3–88.8)	0.97	0.46	0.75	0.92	12.15 (3.78–273.8)	<0.001
Mid lateral	−25	71.0 (57.6–84.4)	0.79	0.56	0.74	0.64	4.93 (1.63–15.0)	0.006
Mid posterior	−25	70.9 (58.3–83.6)	0.76	0.56	0.72	0.61	4.10 (1.38–12.17)	0.015
Mid inferior	−23	75.9 (64.1–87.7)	0.62	0.88	0.89	0.58	11.2 (2.84–44.1)	<0.001
MW index (mmHg%)								
Basal lateral	2200	72.6 (58.2–87.1)	0.85	0.62	0.79	0.71	9.17 (2.76–30.43)	<0.001
Mid lateral	2500	70.0 (52.5–83.1)	0.92	0.46	0.73	0.79	10.15 (2.44–42.24)	<0.001
Mid posterior	2400	66.8 (52.8–80.8)	0.84	0.5	0.73	0.67	5.33 (1.63–17.43)	0.009
Mid inferior	2400	66.1 (52.1–80.2)	0.82	0.5	0.73	0.63	4.57 (1.46–14.35)	0.011

AUC: area under the curve, PPV: positive predictive value, MW index: myocardial work index, NPV: negative predictive value, OR: odds ratio.

**Table 6:** Univariate logistic regression analysis for a combination of peak segmental strain and MW index (for the pre-specified cut-off values) as predictors of the primary end-point.

	OR	*p*
Basal lateral Peak segmental strain −25% + MW index 2200 mmHg%	32.15 (3.78–273.8)	<0.001
Mid lateral Peak segmental strain −25% + MW index 2500 mmHg%	10.43 (2.28–38.93)	<0.001
Mid posterior Peak segmental strain −25% + MW index 2400 mmHg%	6.09 (1.79–20.74)	0.004
Mid inferior Peak segmental strain −22% + MW index 2400 mmHg%	5.50 (1.69–17.93)	0.005

OR: odds ratio, MW: myocardial work.

## Data Availability

The data sets used and/or analyzed during the current study are available from the corresponding author on reasonable request.

## Supplementary Materials

The following supporting information can be downloaded at: https://www.mdpi.com/article/10.3390/jcdd10040181/s1, Supplementary Figure S1: Peak segmental strain comparison between MAD+, MAD- and healthy groups in sixteen segments; Supplementary Figure S2: Myocardial work index comparison between MAD+, MAD− and healthy groups in sixteen segments; Supplementary Video S1: Transthoracic echocardiography (long-axis parasternal view) of MVP patient with MAD presence (systolic separation of the mitral leaflet and left atrial junction from the summit to the left ventricular posterior wall); Supplementary Table S1: The number of strains with abnormally increased peak segmental strain values; Supplementary Table S2: Inter- and Intra-Observer Variability of GLS, peak segmental strains and segmental MW index values.
