# Supplementary material for: Left Ventricle Segmental Longitudinal Strain and Regional Myocardial Work Index Could Help Determine Mitral Valve Prolapse Patients with Increased Risk of Ventricular Arrhythmias
Source: J Cardiovasc Dev Dis. 2023 Apr 20;10(4):181. doi: 10.3390/jcdd10040181 (PMC10145267; doi:10.3390/jcdd10040181)
Supplement: Supplementary file 1 [file jcdd-10-00181-s001.zip › jcdd-2307211 - Supplementary materials.pdf]

Supplementary materials

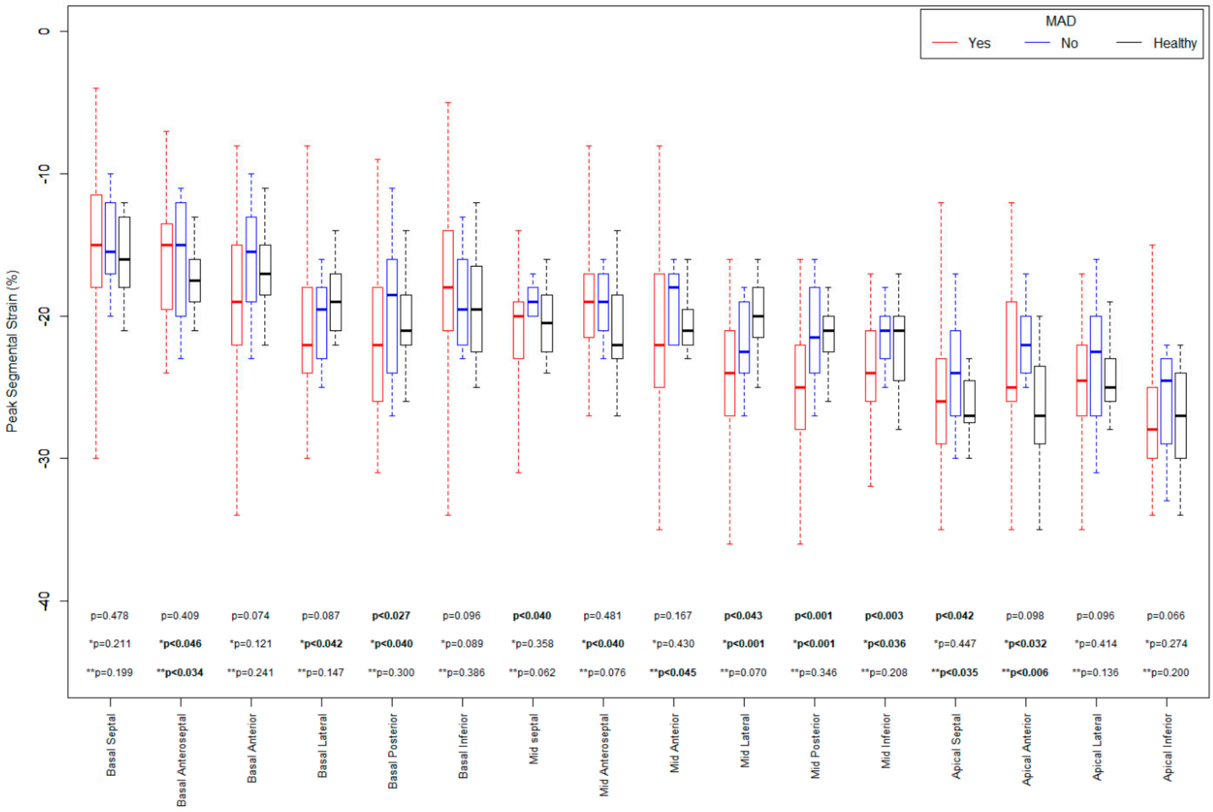

Figure S1. Peak Segmental Strain comparison between MAD+, MAD- and Healthy groups in sixteen segments.

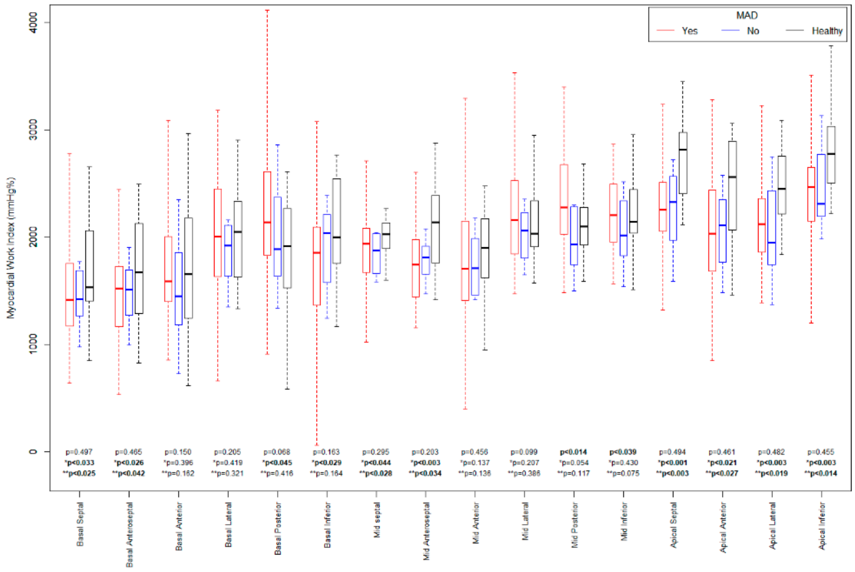

Figure S2. Myocardial Work Index comparison between MAD+, MAD- and Healthy groups in sixteen segments.

Table S1. The number of strains with abnormally increased peak segmental strain values

	MAD+ n = 47	MAD- n = 25	Healthy n = 20	p*	p**	p***
**Peak segmental strain ≥ 25% for all segments**	8 (5 - 9)	3 (1 - 6)	4 (3 - 5)	**0.005**	**0.040**	0.103
**Peak segmental strain ≥ 25% for basal**	2 (1 - 2)	1 (0 - 1)	0 (0 - 0)	**0.014**	**0.004**	0.359
**Peak segmental strain ≥ 25% for basal and mid**	3 (3 - 5)	1 (1 - 3)	0 (0 - 1)	**<0.000**	**<0.000**	0.354

p* - p between MAD+ and MAD-; p** - p between MAD+ and Healthy; p*** - p between MAD- and Healthy

MAD – mitral annular disjunction

Table S2. Inter- and Intra-Observer Variability of GLS, peak segmental strains and segmental MW index values

	Intra - Observer				Inter - Observer			
	Bias	95% Limits of Agreement	ICC	CV (%)	Bias	95% Limits of Agreement	ICC	CV (%)
**Peak segmental strain (%)**	- 0.74	- 0.35 to -1.12	0.87*	17	0.86	0.48 to 1.25	0.83*	17
**MW index (mmHg%)**	119.36	90.86 to 147.86	0.92	14	-88.95	-61.48 to -114.44	0.88	13

*p < 0.001. ICC - intra-class correlation coefficient, CV – coefficient of variation, MW – myocardial work

Video S1. Transthoracic echocardiography (long-axis parasternal view) of MVP patient with MAD presence (systolic separation of the mitral leaflet and left atrial junction from the summit to the left ventricular posterior wall).
